# Bidirectional interconversion of microwave and light with thin-film lithium niobate

**DOI:** 10.1038/s41467-021-24809-y

**Published:** 2021-07-22

**Authors:** Yuntao Xu, Ayed Al Sayem, Linran Fan, Chang-Ling Zou, Sihao Wang, Risheng Cheng, Wei Fu, Likai Yang, Mingrui Xu, Hong X. Tang

**Affiliations:** 1 Department of Electrical Engineering, Yale University, New Haven, CT USA; 2 Present Address: College of Optical Sciences, The University of Arizona, Tucson, AZ USA

**Keywords:** Microwave photonics, Optoelectronic devices and components, Photonic devices, Superconducting devices

## Abstract

Superconducting cavity electro-optics presents a promising route to coherently convert microwave and optical photons and distribute quantum entanglement between superconducting circuits over long-distance. Strong Pockels nonlinearity and high-performance optical cavity are the prerequisites for high conversion efficiency. Thin-film lithium niobate (TFLN) offers these desired characteristics. Despite significant recent progresses, only unidirectional conversion with efficiencies on the order of 10^−5^ has been realized. In this article, we demonstrate the bidirectional electro-optic conversion in TFLN-superconductor hybrid system, with conversion efficiency improved by more than three orders of magnitude. Our air-clad device architecture boosts the sustainable intracavity pump power at cryogenic temperatures by suppressing the prominent photorefractive effect that limits cryogenic performance of TFLN, and reaches an efficiency of 1.02% (internal efficiency of 15.2%). This work firmly establishes the TFLN-superconductor hybrid EO system as a highly competitive transduction platform for future quantum network applications.

## Abstract (web summary)

Coherent conversion between optical and microwave photonics is needed for future quantum applications. Here, the authors combine thin-film lithium niobate and superconductor platforms as a hybrid electro-optic system to achieve high-efficiency frequency conversion between microwave and optical modes.

## Introduction

With superconducting circuits emerging as a promising platform for quantum computation^1–6^ and optical photons being the most suitable long-haul quantum information carrier^7,8^, efficient bidirectional conversion between microwave and optical photons at the quantum level is in critical demand^9–11^. Through high-efficiency electro-optic interface, a hybrid system where quantum information is processed by superconducting circuits and distributed with photonic circuits is one of the most promising schemes to implement large-scale quantum networks^12–16^. Various approaches have realized coherent microwave-to-optical conversion, including spin ensembles^17–20^, electro-optomechanics (EOM)^21–30^, rare-earth-doped crystal^31,32^, ferromagnetic magnons^33,34^, and etc. The highest conversion efficiency of 47% was demonstrated in EOM systems using bulk optical cavity and free-standing megahertz mechanical membranes^25^. However, it is difficult to operate such transducers at the quantum ground state due to the use of low-frequency mechanical resonators.

The cavity electro-optic (EO) system utilizes the Pockels nonlinearity to realize direct conversion between GHz microwave and optical photons^35–37^ without introducing an intermediate excitation. With on-chip frequency tuning, high power handling capability, and robust device design, the highest conversion efficiency for integrated platforms was realized in an aluminum nitride (AlN) cavity EO converter^38^. Recently, bidirectional EO conversion at the microwave ground state has been demonstrated with bulk lithium niobate (LN)^39^ and integrated AlN resonators^40^ in dilution refrigerators at millikelvin temperatures.

While promising results have been achieved in AlN EO devices, the relatively weak Pockels effect of AlN makes the further improvement of the conversion efficiency challenging. To tackle this issue, thin-film LN (TFLN) is a promising candidate because of its strong Pockels nonlinearity^41^. This can lead to a significantly larger vacuum EO coupling rate (*g*_eo_), which has been demonstrated recently^42,43^. Nevertheless, the achieved conversion efficiency is limited to ~ 10^−5^, which is relatively low considering the large EO coefficient of LN. It has also been pointed out that the performance of TFLN is largely limited by the prominent photorefractive (PR) effect which is particularly challenging for cryogenic operations^42–44^. Though it seems not to be a crucial limitation in the bulk LN^39^, the PR effect in TFLN severely constrains the pump power that can be applied in the integrated device. Additionally, the associated charge-screening effect can offset and even cancel the external DC tuning voltage, therefore cause a frequency mismatch between microwave resonance and optical mode splitting^44^. As a result, only unidirectional microwave-to-optical conversion has been realized in TFLN EO converters. The bidirectionality of EO conversion process, which is important for two-way quantum networks, remains to be demonstrated.

In this article, we demonstrate a superconducting-photonic hybrid EO converter based on TFLN optical cavities reaching 1.02% on-chip efficiency and 15.2% internal efficiency. The PR effect and its associated charge-screening effects in TFLN are mitigated by a significant margin through utilizing an air-clad device architecture that eliminates the amorphous oxide buffer layer commonly used as waveguide cladding. This oxide buffer is also employed in previous EO conversion devices to separate superconducting and photonic waveguide structures. The improved efficiency and stability lead to the demonstration of the bidirectional conversion process with TFLN. This buffer-free architecture is also advantageous for future integration with superconducting circuits, where amorphous oxides are undesired for their role in hosting two-level systems^45,46^. The impact of device packaging on the residual RF loss is further examined. We project that with further device improvement in light coupling and cryogenic packaging, the TFLN EO converter could approach unitary internal conversion efficiency, thus enabling a highly competitive transduction platform for future quantum networks.

## Result

### Cavity electro-optics

**Fig. 1:**
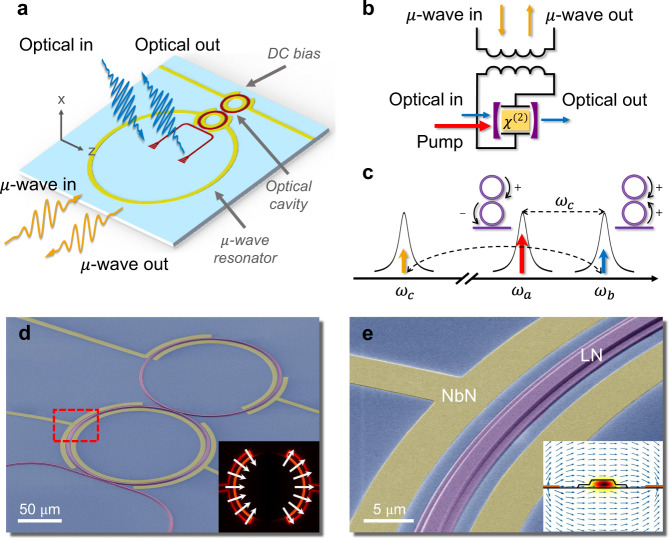
Superconducting cavity electro-optics system and device design. **a** Schematic layout of the TFLN EO converter, where two strongly coupled microring resonators (red) are co-integrated with a planar superconducting resonator (yellow). DC electrodes are utilized for on-chip tuning of the optical modes. **b** Circuit representation of the cavity EO system. The capacitor plate of the lumped-element microwave resonator is coupled to the *χ*^(2)^ optical cavity. **c** Three wave-mixing EO conversion process. The optical doublet corresponds to the symmetric and anti-symmetric supermodes of the coupled microrings. A strong pump (red) at the anti-symmetric mode frequency parametrically stimulates the bidirectional conversion between signal photons in the symmetric mode (blue) and the microwave mode (yellow). **d**, **e** False-color scanning electron microscope images of the EO converter device. Insets show the electric field profiles of interacting optical and microwave modes.

Figure 1a illustrates the schematic layout of our TFLN cavity EO converter, which consists of a pair of strongly coupled ring resonators patterned from x-cut TFLN and a superconducting microwave resonator of niobium nitride (NbN). The microwave resonator capacitively couples to one of the double rings for EO conversion, while a pair of DC electrodes is coupled to the other microring for electrical tuning of the optical resonance modes. The circuit representation of the cavity EO system is illustrated in Fig. 1b, where the electric field of a lumped-circuit LC resonator overlaps with the optical cavity field within the LN EO media through Pockels effect. As a result of the cavity-enhanced EO interaction at the triply-resonant condition, the input microwave field modulates the optical pump and produces an optical sideband at the signal mode frequency. Reversely, a microwave field output can be generated by the optical frequency mixing in the LN cavity.

This triply-resonant enhanced EO conversion scheme, as illustrated in Fig. 1c in the frequency domain, is described by an interaction Hamiltonian *H*_I_ = *ℏg*_eo_(*ab*^†^*c* + *a*^†^*bc*^†^). Here, *a*, *b* and *c* denote annihilation operators for the optical pump, signal, and microwave modes, respectively. *g*_eo_ denotes the vacuum EO interaction strength, which in turn is determined by the mode volume, triple-mode overlap, and the Pockels coefficient. The optical frequency doublet (Fig. 1c) corresponds to the symmetric and anti-symmetric supermodes of the photonic molecule^47^ induced by the strong mutual coupling between the fundamental transverse electric (TE) modes of the double-microring resonators^48^. To leverage the highest EO coefficient *r*_33_ ~ 30 pm/V in x-cut TFLN^41^, a microwave mode with an in-plane electrical field is utilized. The corresponding microwave and optical mode profiles are shown in the insets of Fig. 1d, e.

To initiate frequency conversion, the lower-frequency supermode in the photonic molecule, i.e., the anti-symmetric optical mode *a*, is loaded with a strong pump to produce a photon-number enhanced electro-optical linear conversion. The advantage of the cavity-enhanced coherent conversion is quantified by the cooperativity $C=4{n}_{p}{g}_{{{{{\mathrm{eo}}}}}}^{2}/{\kappa }_{b}{\kappa }_{c}$, where *n*_*p*_ is the intracavity pump photon number and *κ*_*b*_, *κ*_*c*_ are the total energy loss rates for modes *b* and *c*, receptively. By applying a DC bias through the electrode, the frequency splitting of the optical doublet can be tuned to match the microwave resonance frequency, and fulfill the triply-resonant condition (*ω*_*a*_ + *ω*_*c*_ = *ω*_*b*_). Under this ideal condition, the on-chip peak conversion efficiency is given by,

1 $$\eta =\frac{{\kappa }_{b,{{{{\mathrm{ex}}}}}}}{{\kappa }_{b}}\frac{{\kappa }_{c,{{{{\mathrm{ex}}}}}}}{{\kappa }_{c}}\frac{4C}{{(1+C)}^{2}}.$$

Here, *κ*_*b*,ex_ (*κ*_*c*,ex_) is the external loss rates for mode *b*(*c*), and *η*_int_ = 4*C*/(1 + *C*)^2^ is the internal conversion efficiency. Optimal conversion is achieved when *C* = 1, and the corresponding on-chip efficiency is eventually limited by the extraction ratio *κ*_*b*,ex_*κ*_*c*,ex_/*κ*_*b*_*κ*_*c*_.

### Mitigation of the photorefractive effect

**Fig. 2:**
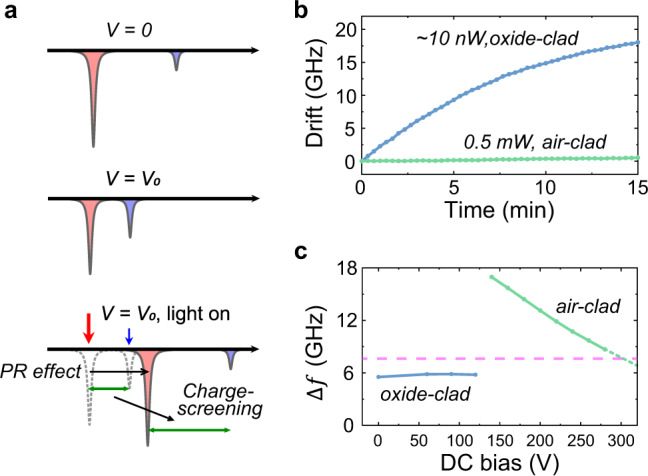
Mitigation of the photorefractive effect. **a** Illustration of the PR-induced resonance instability resulting from a combination of absolute frequency shift and cancellation of voltage tunability after illumination. **b** The measured resonance frequency drift at zero-bias voltage after light illumination of oxide-clad (blue) and air-clad (green) architecture. The drift rate of the measured air-clad device is ${\sim}0.28\ {{{{{\rm{pm/min}}}}}}$ under 0.5 mW optical power in the waveguide. **c** The optical supermode splitting Δ*f* as a function of DC bias for oxide-clad (blue) and air-clad (green) devices. The target microwave frequency is marked as a dashed line.

As indicated by Eq. (1), the EO conversion efficiency *η* increases with the pump photon number. On the TFLN platform, the strong PR effect places a limit on achievable intracavity pump power^42,43^. The PR effect not only destabilizes cavity resonances but also leads to a charge-screening effect which neutralizes the dc-bias field used for tuning the resonances^44^. The PR effect in LN results from a combination of cascaded processes that first builds up a space-charge field in presence of light illumination and subsequently modulates the refractive index of the material^41^. An illustration of the resonance instability is shown in Fig. 2a. Unique to the cryogenic temperatures, the relaxation time of the PR effect is found to be dramatically increased^49^, leading to an accumulated space-charge field that semi-permanently shifts the resonance frequency. Because of the charge accumulation and the strong photovoltaic dynamics, this screening effect could not be compensated for through the regular feedback technique, as the tuning voltage required for phase matching will continuously increase and finally lead to an electrical breakdown of the material.

We found that the PR effect and the charge-screening effect are highly related to the converter design as well as the treatment of LN material. The LN-oxide top-cladding interface is identified to be the major source that aggravates the PR effect^50^. With an air-clad architecture (false-color scanning electron microscope images shown in Fig. 1d, e), the detrimental PR effect and charge-screening effect at cryogenic temperature can be effectively mitigated. In our final converter device, the absolute resonance shift induces by the PR effect is reduced by a significant margin (Fig. 2b). Although a residual drift still exists, a slow PID loop can be utilized to allow the drive laser to follow the resonance frequency during the measurement. A large intracavity photon number can be maintained with stable alignment achieved between the optical supermode splitting Δ*f* and the microwave resonance frequency (Fig. 2c). This air-clad architecture not only enables long-time stable operation of TFLN transduction devices at cryogenic temperatures; it will also benefit future integration of superconducting qubits because the amorphous oxide buffer is known to host the two-level-systems that impact the qubit coherence^45,46^.

### Device fabrication

The optical components of the converter are patterned from 600 nm-thick x-cut TFLN on a sapphire substrate. Optical resonators and waveguides are first defined by electron beam lithography (EBL), then 350 nm of the TFLN is subsequently etched. The majority of the remaining 250 nm TFLN slab is removed in a second EBL and etching step in order to minimize the total coverage of TFLN, leaving only a narrow pedestal to support the microring resonators. This is a crucial step to suppress the microwave loss induced by the strong piezo-electricity of LN. After patterning the optical structures, superconducting NbN is directly deposited on the sapphire substrate using atomic layer deposition and then etched to form the microwave resonator and tuning electrodes. With our air-clad architecture, the superconductor traces are also designed to capacitively couple across the waveguides without climbing over the photonic waveguides, thus significantly reducing the fabrication complexity.

### Bidirectional conversion and efficiency

**Fig. 3:**
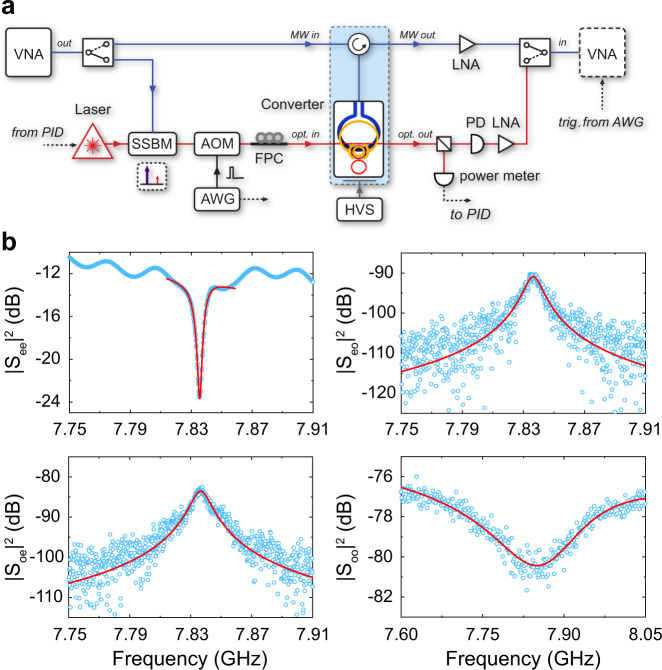
Bidirectional conversion. **a** Measurement setup. The output of a vector network analyzer (VNA) is sent to the device either as the microwave input or as the optical input through a single-sideband modulator (SSBM). For quasi-CW measurements, the optical pump is gated by an acoustic-optic modulator (AOM) driven by an arbitrary wave generator (AWG), which also triggers the VNA. The pump is locked to the optical cavity with a PID controller. FPC: fiber polarization controller. PD: photodetector. LNA: low-noise amplifier. HVS: high voltage source. **b** Measured microwave reflection *S*_ee_, optical-to-microwave conversion *S*_eo_, microwave-to-optical conversion *S*_oe_ and optical reflection *S*_oo_. The peak optical pump power is −12.0 dBm in the waveguide and all matrix coefficients are normalized to the VNA output.

The schematic measurement setup for EO conversion is presented in Fig. 3a. The device is loaded in a closed-cycle refrigerator (Cryomech) cooled down to 1.9 K base temperature. A tunable telecom laser (Santec 710) amplified by an erbium-doped fiber amplifier is tuned to the anti-symmetric mode (lower frequency supermode) as a parametric pump. A pair of grating couplers designed for TE polarized light is used to couple light in and out of the device. One percent of the transmitted light from the device is sampled to provide feedback to the laser wavelength through its build-in piezo. An RF signal from a vector network analyzer (VNA) is either directly sent to the device as the microwave signal input or used to generate the optical signal input through optical single-sideband modulation^38^. The microwave signal is readout wirelessly by terminating a coaxial cable with a hoop antenna^19,40^ which inductively couples to the superconducting resonator. The microwave power delivered to the device is fixed to ~ −50 dBm to avoid the nonlinear response of the superconducting resonator due to its kinetic inductance. The microwave output from the converter is analyzed after a low-noise amplifier at room temperature. The optical output is collected and down-converted through heterodyne with the pump laser by a high-speed photodetector and then sent to the VNA. For measurement in the high power regime (> −2 dBm), the thermal load to the device is reduced by gating the pump light using a high extinction acoustic-optic modulator driven by an arbitrary waveform generator.

With a DC bias of 220 V, the optical mode splitting is tuned (at a rate of ~0.6 pm/V) to match the microwave resonance frequency. Figure 3b shows the full spectra of scattering matrix elements employed for calibrating the conversion efficiency: optical refection *S*_oo_, microwave refection *S*_ee_, microwave-to-optical conversion *S*_oe_ and optical-to-microwave conversion *S*_eo_. The bidirectional nature of the EO conversion is thus fully established. The Lorentzian fits of the *S*_oe_ and *S*_eo_ spectra yield a 3 dB bandwidth matching the microwave linewidth. The peak conversion efficiency is obtained by calibrating out the off-chip gain and loss factor of each input and output signal path^23^:

2 $$\eta =\frac{{S}_{{{{{{\rm{eo,p}}}}}}}{S}_{{{{{{\rm{oe,p}}}}}}}}{{S}_{{{{{{\rm{oo,bg}}}}}}}{S}_{{{{{{\rm{ee,bg}}}}}}}},$$

where *S*_oe,p_ and *S*_eo,p_ are the peaks of conversion spectra, *S*_oo,bg_ and *S*_ee,bg_ are the backgrounds of reflection spectra, respectively.

**Fig. 4:**
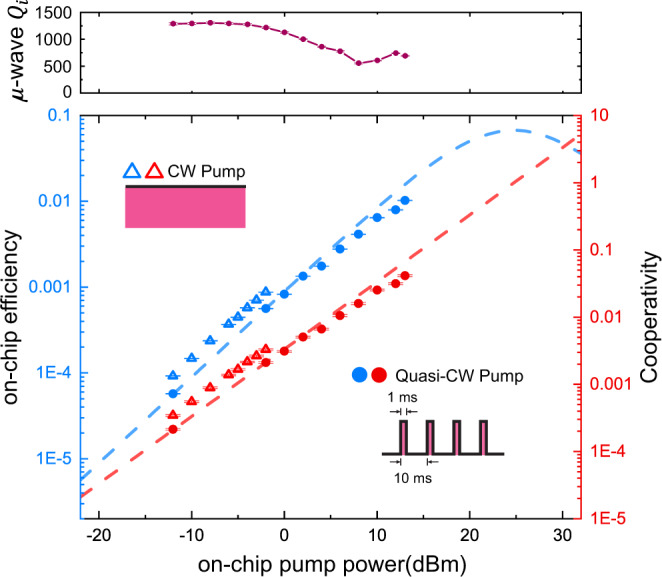
Conversion efficiency. On-chip conversion efficiency (blue) and cooperativity (red) as a function of the peak optical pump power in the input waveguide. The discontinuity in the CW and quasi-CW pump data is due to switching to different pairs of optical modes during the measurements. The maximum conversion efficiency is recorded with a peak pump power of 13.0 dBm. The dashed lines are the theoretical predictions obtained from the data measured with a quasi-CW pump. The measured intrinsic microwave Q under different optical pump powers are presented in the upper panel. The error bars show the standard derivations of the measurements.

The on-chip optical pump power is gradually increased from −12.0 dBm with the conversion efficiency measured at each step using the above-mentioned calibration procedure. Considering the loss and coupling characteristics of microwave and optical cavities, the internal conversion efficiency *η*_int_ and thus cooperativity *C* under different pump power is extracted from Eq. (1), as shown in Fig. 4. At higher pump power levels (> −2.0 dBm), in order to reduce the thermal load to the fridge, we switch to 10% duty cycle quasi-CW pump. After extended exposure during CW measurement, the pair of optical modes we used are no longer accessible due to the accumulated charges. Therefore, we select another pair of optical modes (within the same device) in the quasi-CW measurement, leading to a discontinuity in the data-trace. Detailed device characteristic is presented in Table 1.

**Table 1:** Device parameters.

	Frequency *ω*/2*π*	Total loss rate *κ*/2*π*	External loss rate *κ*_ex_/2*π*
Pump mode *a* (CW)	193.34 THz	301 MHz	109 MHz
Signal mode *b* (CW)	193.35 THz	173 MHz	33 MHz
Pump mode *a* (quasi-CW)	192.83 THz	380 MHz	124 MHz
Signal mode *b* (quasi-CW)	192.84 THz	280 MHz	52 MHz
MW mode *c*	7.836 GHz	9.06 MHz	3.22 MHz

A maximum on-chip conversion efficiency of 1.02 ± 0.01% (internal efficiency of 15.2 ± 0.4%) is recorded with a peak optical pump power adjusted to 13.0 dBm in the waveguide. The corresponding cooperativity reaches a value of 0.041 ± 0.001, which is significantly improved over previously obtained values^42,43^. We note that the cooperativity no longer increases linearly with peak pump power in high power regime. This is attributed to the quality factor degradation of the superconducting resonator, as shown in the upper panel of Fig. 4. From a linear fitting (dashed lines) in Fig. 4 of the conversion efficiency in the low power regime, the vacuum coupling rate is estimated to be *g*_eo_ = 2*π* × 750 Hz, which is within the same order of magnitude compared to the simulated value (2*π* × 1.5 kHz)^38^.

## Discussion

**Fig. 5:**
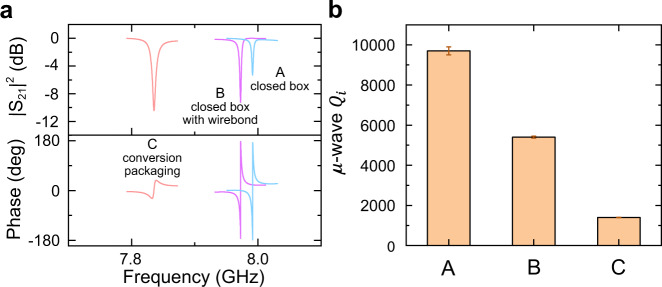
Superconducting microwave cavity performance. **a** Microwave reflection spectrum of the device (A) in a closed copper box (B) in a closed copper box with wire bonds (C) packaged with fiber-array for conversion measurement. **b** The fitted intrinsic microwave quality factors. The error bars show the standard derivations of the measurements.

The most obvious route to increase the conversion efficiency is improving the microwave quality factor of the current device. In particular, we find that the microwave *Q* is significantly affected by how the device is assembled for cryogenic operation. When the TFLN integrated device is characterized in an RF-tight, closed copper box package which suppresses the undesired microwave modes from coupling to the on-chip resonator, the device exhibits an intrinsic *Q* of 9.7 × 10^3^ at 2.6 K, with the microwave reflection spectrum shown in Fig. 5 (trace A). The measured quality factor of the microwave resonator coupled to TFLN shows a significantly higher value compared to prior works^42,43^. Furthermore, we notice that the wire bonds used for dc-tuning further contribute to microwave loss. As indicated by trace B shown in Fig. 5, when measured with wire bonds in the same closed box at 2.6 K, the intrinsic Q is reduced by almost a half to 5.4 × 10^3^.

With the final EO conversion package, additional microwave loss arises from the cryogenic optical interfaces required to accommodate the fiber array. The fiber array is manipulated by a set of attocubes for precision alignment, preventing the use of a fully closed copper box. A copper box with an open lid is utilized instead, similar to the configuration previously used in AlN microwave-to-optics experiment^38^ where we did not observe significant *Q* change before and after packaging. However, this is not the case for the TFLN converter studied here. As shown in Fig. 5 (trace C), the device exhibits a further degradation of microwave quality factor, and the extracted intrinsic *Q* drops to 1.3 × 10^3^. Since the only difference compared to the previous measurements is the packaging configuration, we conjecture that the excess microwave loss is mainly induced by the stronger piezoelectric coupling of LN (than AlN) to spurious modes supported by the RF packaging. To mitigate this loss, it is critical to further reduce the TFLN trace volume that does not participate in the EO conversion, and utilize a RF packaging that can effectively suppress spurious modes. The *Q* of 9.7 × 10^3^ measured in the closed box is most likely already limited by the dielectric and piezoelectric coupling loss induced by the residual TFLN traces supporting optical waveguides^42^.

It is anticipated that unitary internal conversion efficiency (*C* = 1) can be obtained at the current pump power level, if the intrinsic *Q* of ~10^4^ can be recovered with improved cryogenic packaging to suppress parasitic losses. Also, the on-chip efficiency could be further improved by engineering the external loss rate of optical and microwave modes. Our current devices use grating couplers which introduce an insertion loss as high as 12.0 dB per facet due to weak index contrast between LN and the sapphire substrate. By utilizing side-coupled inverted tapers, the insertion loss could be reduced to 3.4 dB per facet^40,51^, which will also dramatically reduce the thermal-optic heating and light absorption induced by scattered light.

We note that the microwave resonator is not at its ground state (thermal photon occupancy ${\bar{n}}_{{{{{{\rm{th}}}}}}} \sim 5$) in this work. Characterization of ground state conversion will be done either in a dilution refrigerator at millikelvin temperature^40^ or through a radiative cooling technique with improved microwave *Q*^52^. After that, it is feasible to utilize TFLN-based devices for entanglement distribution via the heralded entanglement generation between microwave and optical photons with blue-detuned pump pulses and photon-counting setup^53,54^.

In conclusion, we demonstrate a cavity EO converter based on TFLN with a conversion efficiency up to 1.02%, which represents a significant improvement over previous works. Utilizing an air-clad architecture to mitigate the PR effect, the device maintains stable operation while supporting high pump photons numbers at cryogenic temperatures. This not only allows us to demonstrate bidirectional conversion with high efficiency, but also points to the possible use of cryogenic TFLN devices for quantum applications such as verification of non-classical correlation, generation of entangled photon pairs etc.^16,53,54^, which all require an extensive stable operation. The system efficiency of the converter can be further improved by proper cryogenic packaging and light coupling. With these improvements, we anticipate achieving high conversion efficiency with sufficiently low pump power for ground state operation at millikelvin temperature for critical quantum network demonstrations.

## Data Availability

The data that support the plots within this paper and other findings of this study are available from the corresponding author (H. X. T.) upon reasonable request.
